# A Conceptual Framework for Engaged Communication at Adult Day Services: A Mixed-Methods Study

**DOI:** 10.1093/geroni/igaa057.689

**Published:** 2020-12-16

**Authors:** Sara Mamo, Kara Wheeler, Olivia Perry

**Affiliations:** 1 University of Massachusetts Amherst, AMHERST, Massachusetts, United States; 2 University of Massachusetts Amherst, Amherst, Massachusetts, United States

## Abstract

Adult Day Services provide an opportunity for social engagement for older adults who might otherwise become isolated. Communication environments at many Adult Day Centers can be difficult for participants due to the high prevalence of hearing loss and poor acoustics in large activity rooms. The purpose of this study is to understand the hearing and social health status of participants across multiple group care settings as well as participants’ challenges and motivations to engage in social communication. A mixed methods approach was undertaken. Seventy-two participants from two Programs for All-inclusive Care for the Elderly (PACE®) completed quantitative measures: hearing test, cognitive screener (MOST™), IOM Social and Behavioral Determinants of Health, UCLA Loneliness Scale, and Instrumental Activities of Daily Living. Using maximum variation sampling based on hearing status and UCLA loneliness scores, ten participants were invited to complete one-on-one semi-structure interviews. Interviews aimed to learn more about how and why participants did and/or did not engage in social communication with other PACE participants. Transcripts were coded and thematic analysis was used to identify common barriers and motivations to communication. A conceptual framework was developed by integrating quantitative and qualitative findings to recognize what contributes to meaningful interactions or engaged communication. The findings from this study will contribute to the development of an intervention to address hearing loss and support communication for older adults in group care settings. In order to maximize the potential benefit of attending group-based day services, the communication barriers and motivations of older adults need to be addressed.

